# Artificial oocyte activation using Ca^2+^ ionophores following intracytoplasmic sperm injection for low fertilization rate

**DOI:** 10.3389/fendo.2023.1131808

**Published:** 2023-03-09

**Authors:** Kazuhiro Akashi, Mitsutoshi Yamada, Seung Chik Jwa, Hiroki Utsuno, Shintaro Kamijo, Yasushi Hirota, Mamoru Tanaka, Yutaka Osuga, Naoaki Kuji

**Affiliations:** ^1^Department of Obstetrics and Gynecology, Keio University School of Medicine, Tokyo, Japan; ^2^Department of Obstetrics and Gynecology, Saitama Medical University, Saitama, Japan; ^3^Clinical Laboratory, Keio University Hospital, Tokyo, Japan; ^4^Department of Obstetrics and Gynecology, Faculty of Medicine, The University of Tokyo, Bunkyo, Tokyo, Japan; ^5^Department of Obstetrics and Gynecology, Tokyo Medical University, Tokyo, Japan

**Keywords:** artificial oocyte activation (AOA), fertilization failure, intracytoplasmic sperm injection (ICSI), infertility, live birth

## Abstract

This large multi-center retrospective study examined whether artificial oocyte activation (AOA) using Ca^2+^ ionophore following ICSI improves the live birth rate for couples with previous ICSI cycles of unexplained low fertilization rate. In this large-scale multi-center retrospective study conducted in Japan, data were collected from Keio University and 17 collaborating institutions of the Japanese Institution for Standardizing Assisted Reproductive Technology. Between January 2015 and December 2019, 198 couples were included in this study. Oocytes for both the intervention and control groups were procured from the same pool of couples. Oocytes obtained from ICSI cycles with no or low fertilization rate (<50%) with unknown causes were included in the control (conventional ICSI) group while oocytes procured from ICSI cycles followed by performing AOA were assigned to the intervention (ICSI-AOA) group. Those fertilized with surgically retrieved sperm were excluded. ICSI-AOA efficacy and safety were evaluated by comparing these two groups. Live birth rate was the primary outcome. The ICSI-AOA group (2,920 oocytes) showed a significantly higher live birth per embryo transfer rate (18.0% [57/316]) compared to that of the conventional ICSI group with no or low fertilization rate (1,973 oocytes; 4.7% [4/85]) (odds ratio 4.5, 95% confidence interval 1.6–12.6; P<0.05). A higher live birth rate was observed in younger patients without a history of oocyte retrieval. Miscarriage, preterm delivery, and fetal congenital malformation rates were similar between the two groups. ICSI-AOA may reduce fertilization failure without increasing risks during the perinatal period. AOA may be offered to couples with an ICSI fertilization rate < 50%.

## 1 Introduction

The introduction of intracytoplasmic sperm injection (ICSI) in 1992 revolutionized the treatment of male infertility, enabling couples facing infertility issues due to severely impaired sperm characteristics to have children (1). Currently, the fertilization rate after ICSI reportedly exceeds 65% (2), though total fertilization failure occurs in 1–3% of all ICSI cycles (3, 4).

Oocyte activation deficiency (OAD) is considered to be a major cause of fertilization failure, accounting for 40–70% of the causes of fertilization failure after ICSI (4–6). In normal fertilization, phospholipase C zeta is released into the oocyte when the sperm enters the oocyte, causing Ca^2+^ oscillation in the oocyte to activate it, which results in fertilization. Meanwhile, if Ca^2+^ oscillation does not occur due to either oocyte- or sperm-related factors, inadequate oocyte activation leads to fertilization failure. At least 68% of OAD is caused by sperm-related factors (7). In contrast, other studies using the mouse oocyte activation test (MOAT) have indicated that only about 16% to 18% of OAD is caused by sperm-related factors alone, suggesting that the majority of OAD is attributable to oocyte-related factors alone or in combination with sperm-related factors (8, 9).

To overcome fertilization failure after ICSI, artificial oocyte activation (AOA) has been developed. Although the efficacy and safety of AOA have not yet been established, AOA is widely performed in clinical practice with a variety of techniques, such as the use of Ca^2+^ ionophores (e.g., ionomycin and calcimycin [A23187]) and strontium chloride (SrCl_2_), mechanical stimulation, and electrical stimulation (10). Among the AOA methods, oocyte activation with SrCl_2_ is considered to be the most invasive, with a high frequency of oocyte degeneration (11–14). In contrast, Ca^2+^ ionophores are expected to cause minimal damage to oocytes (11). In a survey conducted by the Ministry of Health, Labour and Welfare in Japan, 30.8% of facilities report the use of Ca^2+^ ionophores for AOA (15). AOA protocols throughout facilities are diverse with respect to the ionophore concentration and exposure duration, the timing of ionophore exposure following ICSI, and the number of exposure settings (16).

Therefore, to establish the efficacy and safety of AOA using Ca^2+^ ionophores, it is necessary to examine the reagents used and the protocol in detail. To answer these questions, we compared AOA using Ca^2+^ ionophores following ICSI, with ICSI alone by conducting a multicenter retrospective cohort study of couples with a fertilization rate of ≤ 50% after ICSI, identifying live birth rate as the primary outcome.

## 2 Materials and methods

### 2.1 Study design

This retrospective multicentre study was conducted by Keio University with support from 17 collaborating institutions affiliated with the Japanese Institution for Standardizing Assisted Reproductive Technology. We collected the clinical data of all couples who underwent AOA between January 2015 and December 2019 at the participating facilities.

### 2.2 Participants

Oocytes of couples whose fertilization rate with conventional ICSI was < 50% at the last oocyte retrieval and those who subsequently underwent oocyte aspiration with AOA using Ca^2+^ ionophores were considered eligible for the analysis. Thus, oocytes for both the intervention and control groups were retrieved from different cycles of the same pool of couples. Oocytes obtained from conventional ICSI cycles were included in the control (conventional ICSI) group while oocytes procured from ICSI cycles followed by performing AOA were assigned to the intervention (ICSI-AOA) group. As of now, a consensus about the definition of fertilization failure has not yet been established. Although the Vienna consensus defined an 80% fertilization rate after ICSI as the benchmark value (2), we set a cut-off value of < 50% fertilization rate after referring to previous papers (17, 18). The exclusion criteria were as follows: oocyte retrieval cycles with less than two oocytes retrieved; fertilization rate > 50%; a case of microsurgical epididymal sperm aspiration or testicular sperm extraction; women aged over 42 years at the time of initial visit; other AOA methods such as electrical stimulation or SrCl_2_; and cases of two-step embryo transfer (ET).

### 2.3 Setting and definition of primary and secondary outcomes

The primary outcome in this study was live birth rate (number of live births/ET cycles). Secondary outcomes were defined as follows: number of retrieved oocytes; number of retrieved matured oocytes (number of metaphase II [MII] oocytes at the time of stripping); fertilization rate (number of embryos with two pronuclei and two polar bodies within 24 h after ICSI/number of MII oocytes that were used for ICSI); embryo cleavage rate (number of eight-cell stage embryos on day three/number of normally fertilized oocytes); developmental rate of blastocyst embryo (number of blastocysts on day five/number of normally fertilized oocytes); developmental rate of good blastocyst embryos (number of good blastocysts on day five/number of normally fertilized oocytes); degeneration rate (number of oocytes damaged or degenerated after ICSI/number of oocytes that were used for ICSI); ET cancel rate (number of cycles that did not reach fresh ET or embryo freezing/number of cycles in which at least two oocytes were obtained); biochemical pregnancy rate (number of ET cycles with positive pregnancy response/number of ET cycles); clinical pregnancy rate (ET cycle in which the gestational sac is found in the uterus by ultrasound computed tomography/number of ET cycles); miscarriage rate (number of miscarriages/number of clinical pregnancy); preterm birth rate (number of preterm births/number of clinical pregnancies); and congenital malformation rate (number of congenital malformations/number of clinical pregnancies). The embryo cleavage rate was determined 68 ± 1 h after ICSI; the number of blastocysts on day five was determined 116 ± 2 h after ICSI; and the number of good blastocysts was determined as Stage 3 or higher according to Gardner’s classification (19), not including grade C inner cell mass or trophectoderm.

### 2.4 Ovarian stimulation

An ovarian stimulation protocol was selected and performed according to each institution’s criteria. The stimulation protocols were classified into the following seven categories: natural cycle, clomiphene citrate (CC), human menopausal gonadotropin/recombinant follicle-stimulating hormone with CC, gonadotropin-releasing hormone (GnRH) agonist, GnRH antagonist, progestin-primed ovarian stimulation, and other protocols. Both fresh and freeze-thaw ET cycles were included.

### 2.5 AOA procedure

AOA was performed according to each institution’s protocol. All retrieved oocytes were incubated with a solution containing Ca^2+^ ionophores (A23187 or ionomycin) within 30 min after ICSI. The following information about the AOA protocol was obtained from each institution: type of reagent used, timing of AOA administration after ICSI, reagent concentration, and exposure time.

### 2.6 Statistical analysis

The primary and secondary outcomes were compared between the ICSI-AOA and conventional ICSI groups to examine the embryological and clinical outcomes of AOA. In a subgroup analysis, both groups were compared according to further classifications according to the women’s age, history of oocyte retrieval, and fertilization rate of the previous ICSI. The chi-square test, Fisher’s exact test, and Student’s t-test were used for comparison among variables. Odds ratios (ORs) were obtained from a generalized estimating equation model with binomial response and a log link, assuming independent working correlation. The results were adjusted for the other covariates (female age, BMI, history of live births, history of smoking, indication of ART) by using multiple logistic regression analysis. Adjusted odds ratios were also calculated by multiple logistic regression analysis. Statistical analyses were performed using the IBM SPSS Statistics software, version 28 (IBM, Armonk, NY, USA), and GraphPad Prism 9 (GraphPad Software, Inc., La Jolla, CA, USA), as appropriate. *P-*values < 0.05 were considered to be statistically significant.

### 2.7 Ethical approval

This study was approved by the institutional research ethics board of Keio University School of Medicine (approval number: 20211097). The collaborating institutions received approval to participate in the study from their own institutional ethics committees. The need for informed consent was waived by the institutional research ethics board owing to the retrospective nature of the study. Opt-out options were provided for the participants through the website of the Department of Obstetrics and Gynaecology, Keio University School of Medicine, and each collaborating institution.

### 2.8 Role of the funding source

The funders of the study had no role in study design, data collection, data analysis, data interpretation, or writing of the report. The funders-approved committee members discussed the interpretation of the results with the authors.

## 3 Results

### 3.1 Participant characteristics

**Figure 1:**
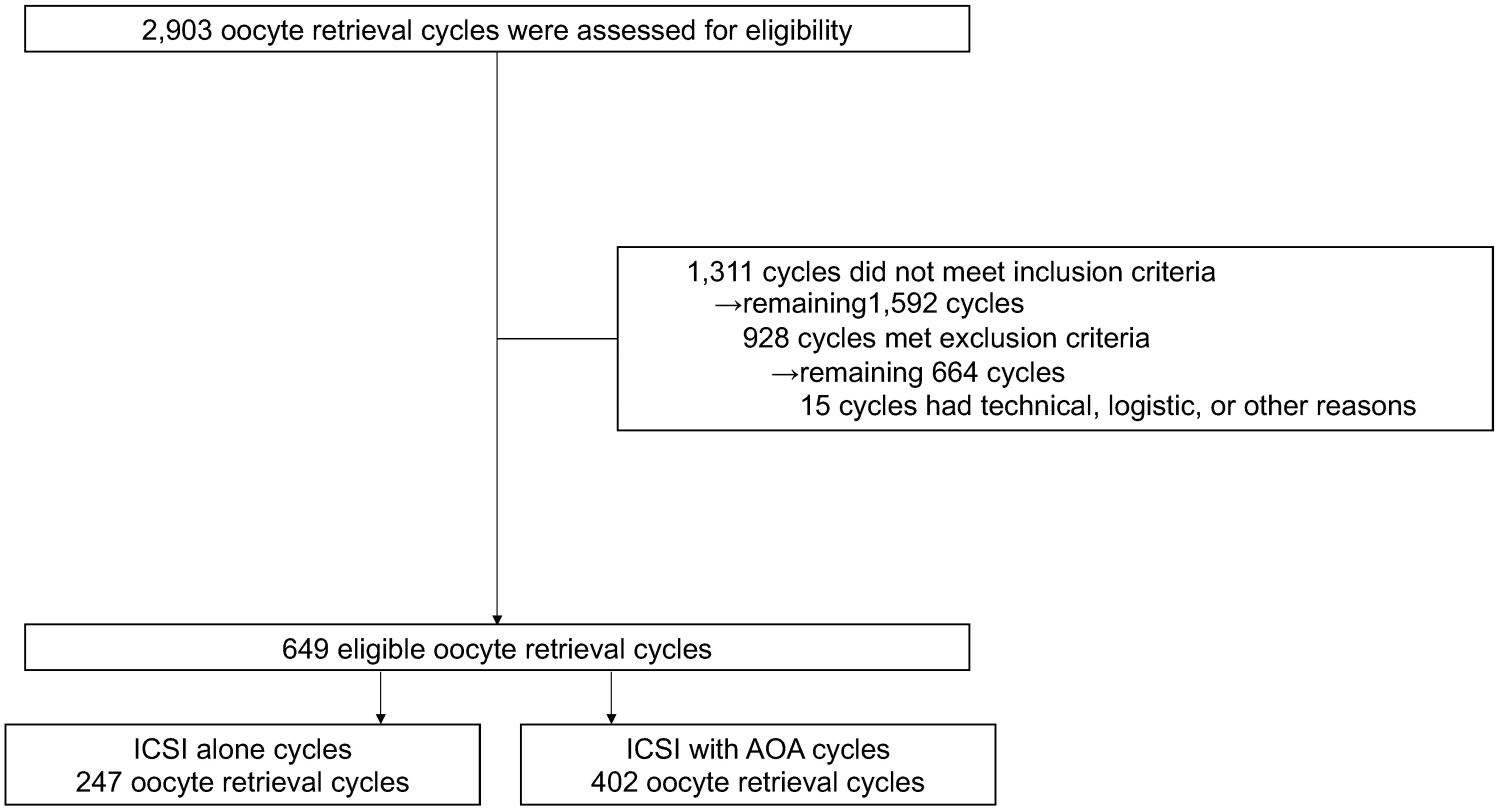
Study flow diagram.

The data of 15,212 oocytes (826 couples and 2,903 oocyte retrieval cycles) were collected from 18 institutions. A total of 4,893 oocytes (198 couples and 649 oocyte retrieval cycles) were finally included in the analysis (**Figure 1**). All embryos were used for frozen-thawed ETs; no fresh ETs were performed. Although no restrictions were placed on the method of freezing, all embryos were frozen by vitrification. The average age (± standard deviation) of women at the initial visit was 35.32 ± 0.27 years, and the average infertility duration was 38.28 ± 3 months (**Supplementary Table 1**). Female age at the time of oocyte retrieval was significantly higher in the ICSI-AOA group (37.74 ± 0.23 years vs. 36.72 ± 0.24 years, *P<*0.001), and the number of mature oocytes was significantly lower in the ICSI-AOA group (4.82 ± 0.26 vs. 5.70 ± 0.27, *P<*0.05) (**Table 1**).

**Table 1:** Embryological and clinical outcomes after intracytoplasmic sperm injection (ICSI) and subsequent artificial oocyte activation (AOA) treatment.

	Conventional ICSI	ICSI-AOA	*P*-value	Odds ratio (95% CI)	Adjusted Odds ratio (95% CI)
**ICSI cycles**	247	402	NA	NA	NA
**Female age at the oocyte retrieval (years)**	36.7 ± 3.9	37.7 ± 3.7	<0.001	NA	NA
**FSH/hMG dose (IU)**	2579.8 ± 2456.8	2238.0 ± 1419.5	NS	NA	NA
**Oocytes retrieved**	8.0 ± 5.7	7.3 ± 5.8	NS	NA	NA
**Matured oocytes**	5.7 ± 4.3	4.8 ± 4.2	<0.05	NA	NA
Fertilization rate (%)*	20.8 (285/1372)	53.7 (1016/1893)	<0.001	4.4 (3.8–5.2)	1.6 (1.3–1.9)
Developmental rate of good cleavage stage embryos (%)**	22.1 (63/285)	36.9 (375/1016)	<0.001	2.1 (1.5–2.8)	1.2 (0.9–1.6)
Developmental rate of blastocyst embryos (%)**	17.2 (49/285)	30.1 (306/1016)	<0.001	2.1 (1.5–2.9)	2.0 (1.4–2.8)
Developmental rate of good blastocyst embryos (%)**	6.3 (18/285)	12.3 (125/1016)	<0.01	2.1 (1.2–3.5)	2.5 (1.5–4.2)
Degeneration rate (%)*	13.3 (183/1372)	11.7 (221/1893)	NS	0.9 (0.7–1.1)	0.9 (0.8–1.1)
**ET cycles**	85	316	NA	NA	NA
ET cancellation rate (%)***	57.5 (142/247)	25.6 (103/402)	<0.001	0.3 (0.2–0.4)	0.2 (0.2–0.3)
Biochemical pregnancy rate (%)****	11.8 (10/85)	35.4 (112/316)	<0.001	4.1 (2.0–8.3)	2.6 (1.4–4.9)
Clinical pregnancy rate (%)****	11.8 (10/85)	28.2 (89/316)	<0.01	2.9 (1.5–5.9)	3.6 (1.7–7.7)
Live birth rate (%)****	4.7 (4/85)	18.0 (57/316)	<0.01	4.5 (1.6–12.6)	5.2 (1.8–15.6)
Miscarriage rate (%)*****	60.0 (6/10)	29.2 (26/89)	NS	1.2 (0.5–3.0)	NA
Preterm birth rate (%)*****	0.0 (0/10)	6.7 (6/89)	NS	NA	NA
Congenital malformation rate (%)*****	0.0 (0/10)	2.1 (1/89)	NS	NA	NA

Values are presented as mean ± standard deviation or % (n/total), unless otherwise indicated. Statistical significance is set at P<0.05.

FSH, follicle-stimulating hormone; hMG, human menopausal gonadotropin; ET, embryo transfer; NS, not significant; NA, not applicable; CI, confidence interval.

* per number of MII oocytes that were used for ICSI; ** per number of normally fertilized oocytes; *** per number of cycles in which at least two oocytes were obtained; **** per number of ET cycles; ***** per number of clinical pregnancies.

### 3.2 Embryological and clinical outcomes including live birth after AOA

The fertilization rate was significantly higher in the ICSI-AOA group (53.7% [1016/1893]) than in the conventional ICSI group (20.8% [285/1372]) (OR 4.4, 95% confidence interval [CI] 3.8–5.2; *P*<0.001). The ICSI-AOA group had significantly higher developmental rates of good cleavage stage embryos, blastocysts, and good blastocysts per fertilized embryos (36.9% [375/1016], 30.1% [306/1016], and 12.3% [125/1016], respectively) compared with the conventional ICSI group (22.1% [63/285], 17.2% [49/285], and 6.3% [18/285], respectively) (OR 2.1, 95% CI 1.5–2.8, *P*<0.001; OR 2.1, 95% CI 1.5–2.9, *P*<0.001; and OR 2.1, 95% CI 1.2–3.5, *P*<0.01, respectively).

The biochemical and clinical pregnancy rates per ET cycle were significantly higher in the ICSI-AOA group (35.4% [112/316] and 28.2% [89/316], respectively) compared with those in the conventional ICSI group (11.8% [10/85] and 11.8% [10/85], respectively) (OR 4.1, 95% CI 2.0–8.3, *P*<0.001 and OR 2.9, 95% CI 1.5–5.9, *P*<0.01, respectively). Overall, ICSI-AOA treatment significantly increased the live birth rate (18.0% [57/316]) compared with ICSI alone (4.7% [4/85]) (OR 4.5, 95% CI 1.6–12.6; *P*<0.01). ICSI-AOA treatment also significantly decreased the ET cancellation rate (25.6% [103/402]) compared with ICSI alone (57.5% [142/247]) (OR 0.3, 95% CI 0.2–0.4; *P*<0.001). The rates of oocyte degeneration, miscarriage, preterm delivery, and fatal congenital anomalies were similar between the two groups. After adjusted by multiple regression analysis, similar significant differences were confirmed for these outcomes.

The ovarian stimulation protocol during the oocyte retrieval cycle varied between couples, as well as the cycles; however, there were no significant differences in clinical pregnancy or live birth rates among the ovarian stimulation protocols (**Supplementary Table 2**).

Nine protocols of AOA were used at each institution in terms of the type of reagents, timing of AOA after ICSI, concentration of the reagent, and drug exposure time. No significant differences in clinical pregnancy and live birth rates were observed among the protocols Either A23187 or ionomycin was used in each institution. They are known to differ in length and strength with respect to Ca^2+^ release, which can lead to heterogeneity between protocols (20). The total 316 transfer cycles following ICSI-AOA were classified into two groups: the A23187 group and Ionomycin group. The clinical pregnancy and live birth rates were compared, with no significant differences observed between the two groups (**Supplementary Table 3**).

### 3.3 Populations that benefit from ICSI-AOA

It is known that *in vitro* fertilization results are better in younger age groups (21). Accordingly, we evaluated the effectiveness of ICSI-AOA treatment by dividing the couples into two groups according to their age at the first visit: a younger (≤ 35 years) and an older group (≥ 36 years) (**Table 2**). In the younger group, the ICSI-AOA subgroup had significantly higher clinical pregnancy (32.1% [52/162]) and live birth (19.1% [31/162]) rates compared with those in the conventional ICSI subgroup (5.9% [3/51] and 0.0% [0/51], respectively) (OR 7.6, 95% CI 2.3–25.4, *P*<0.001; OR not applicable [NA], 95% CI NA, *P*<0.001), while no differences were observed in the older subgroup.

**Table 2:** Embryological and clinical outcomes after intracytoplasmic sperm injection (ICSI) and subsequent artificial oocyte activation (AOA) treatment according to patient age.

	Female age at the oocyte retrieval: ≤ 35 years				Female age at the oocyte retrieval: 36–41 years			
	Conventional ICSI	ICSI-AOA	*P*-value	Odds ratio (95% CI)	Conventional ICSI	ICSI-AOA	*P*-value	Odds ratio (95% CI)
**ICSI cycles**	115	195	NA	NA	132	207	NA	NA
**Female age at the oocyte retrieval (years)**	34.1 ± 3.8	35.2 ± 3.6	<0.05	NA	39.0 ± 2.0	40.1 ± 1.6	<0.001	NA
**FSH/hMG dose (IU)**	2294.5 ± 1164.8	2246.8 ± 1266.8	NS	NA	2833.3 ± 3171.6	2229.8 ± 1547.1	NS	NA
**Oocytes retrieved**	9.1 ± 5.9	8.3 ± 6.2	NS	NA	7.0 ± 5.4	6.3 ± 5.2	NS	NA
**Matured oocytes**	6.4 ± 4.4	5.4 ± 4.5	<0.05	NA	5.1 ± 4.1	4.3 ± 3.7	NS	NA
Fertilization rate (%)*	23.5 (170/722)	53.0 (549/1036)	<0.001	3.7 (3.0–4.5)	17.7 (115/650)	54.5 (467/857)	<0.001	5.6 (4.3–7.1)
Developmental rate of good cleavage stage embryo (%)**	21.8 (37/170)	34.6 (190/549)	<0.01	1.9 (1.3–2.9)	22.6 (26/115)	39.6 (185/467)	<0.001	2.2 (1.4–3.6)
Developmental rate of blastocyst embryo (%)**	15.9 (27/170)	27.5 (151/549)	<0.01	2.0 (1.3–3.2)	19.1 (22/115)	33.2 (155/467)	<0.01	2.1 (1.3–3.5)
Developmental rate of good blastocyst embryo (%)**	7.1 (12/170)	12.9 (71/549)	<0.05	2.0 (1.0–3.7)	5.2 (6/115)	11.6 (54/467)	<0.05	2.4 (1.0–5.7)
Degeneration rate (%)*	11.8 (85/722)	12.4 (128/1036)	NS	1.1 (0.8–1.4)	15.1 (98/650)	10.9 (93/857)	<0.05	0.7 (0.5–0.9)
**ET cycles**	51	162	NA	NA	34	154	NA	NA
ET cancellation rate (%)***	47.0 (54/115)	25.1 (49/195)	<0.001	0.4 (0.2–0.6)	66.7 (88/132)	26.1 (54/207)	<0.001	0.2 (0.1–0.3)
Biochemical pregnancy rate (%)****	5.9 (3/51)	35.8 (58/162)	<0.001	8.9 (2.7–29.9)	20.6 (7/34)	35.1 (54/154)	NS	2.1 (0.8–5.1)
Clinical pregnancy rate (%)****	5.9 (3/51)	32.1 (52/162)	<0.001	7.6 (2.3–25.4)	20.6 (7/34)	24.0 (37/154)	NS	1.2 (0.5–3.0)
Live birth rate (%)****	0.0 (0/51)	19.1 (31/162)	<0.001	NA	11.8 (4/34)	16.9 (26/154)	NS	1.5 (0.5–4.7)
Miscarriage rate (%)*****	100.0 (3/3)	30.8 (16/52)	NA	NA	47.9 (3/7)	27.0 (10/37)	NA	NA
Preterm birth rate (%)*****	0.0 (0/3)	5.8 (3/52)	NA	NA	0.0 (0/7)	8.1 (3/37)	NA	NA
Congenital malformation rate (%)*****	0.0 (0/3)	0.0 (0/52)	NA	NA	0.0 (0/7)	2.7 (1/37)	NA	NA

Values are presented as mean ± standard deviation or % (n/total), unless otherwise indicated. Statistical significance is set at P<0.05.

FSH, follicle-stimulating hormone; hMG, human menopausal gonadotropin; ET, embryo transfer; NS, not significant; NA, not applicable; CI, confidence interval.

* per number of MII oocytes that were used for ICSI; ** per number of normally fertilized oocytes; *** per number of cycles in which at least two oocytes were obtained; **** per number of ET cycles; ***** per number of clinical pregnancies.

When oocytes were divided based on the couples’ history of oocyte retrieval (**Table 3**), ICSI-AOA treatment increased the live birth rate from 6.1% (4/66) to 18.4% (49/266) in oocytes from couples without a history of oocyte retrieval (OR 3.5, 95% CI 1.2–10.0; *P*<0.05) and from 0.0% (0/19) to 16.0% (8/50) (not significant [NS]) in those from couples with a history of at least one oocyte retrieval.

**Table 3:** Embryological and clinical outcomes after intracytoplasmic sperm injection (ICSI), and subsequent artificial oocyte activation (AOA) treatment, distributed based on the history of oocyte retrievals.

	History of oocyte retrievals: 0				History of oocyte retrievals: ≥1			
	Conventional ICSI	ICSI-AOA	*P*-value	Odds ratio (95% CI)	Conventional ICSI	ICSI-AOA	*P*-value	Odds ratio (95% CI)
**ICSI cycles**	173	285	NA	NA	74	117	NA	NA
**Female age at the oocyte retrieval (years)**	36.2 ± 4.1	37.7 ± 3.9	<0.001	NA	38.0 ± 2.7	38.0 ± 3.1	NS	NA
**FSH/hMG dose (IU)**	2575.0 ± 2842.1	2196.3 ± 1465.0	NS	NA	2590.5 ± 1211.1	2334.6 ± 1302.9	NS	NA
**Oocytes retrieved**	8.6 ± 6.1	7.5 ± 5.9	<0.05	NA	6.6 ± 4.4	6.8 ± 5.4	NS	NA
**Matured oocytes**	6.0 ± 4.6	5.0 ± 4.1	<0.05	NA	5.0 ± 3.3	4.5 ± 4.2	NS	NA
Fertilization rate (%)*	21.1 (215/1019)	54.5 (742/1362)	<0.001	4.5 (3.7–5.4)	19.8 (70/353)	51.6 (274/531)	<0.001	4.3 (3.2–5.9)
Developmental rate of good cleavage stage embryo (%)**	22.8 (49/215)	39.2 (291/742)	<0.001	2.2 (1.5–3.1)	20.0 (14/70)	30.7 (84/274)	NS	1.8 (0.9–3.4)
Developmental rate of blastocyst embryo (%)**	18.1 (39/215)	31.8 (236/742)	<0.001	2.1 (1.4–3.1)	14.3 (10/70)	25.5 (70/274)	<0.05	2.1 (1.0–4.2)
Developmental rate of good blastocyst embryo (%)**	5.6 (12/215)	13.7 (102/742)	<0.01	2.7 (1.5–5.0)	8.6 (6/70)	8.4 (23/274)	NS	1.0 (0.4–2.5)
Degeneration rate (%)*	12.4 (126/1019)	10.8 (147/1362)	NS	0.9 (0.7–1.1)	16.1 (57/353)	13.9 (74/531)	NS	0.8 (0.6–1.2)
**ET cycles**	66	266	NA	NA	19	50	NA	NA
ET cancellation rate (%)***	54.3 (94/173)	23.5 (67/285)	<0.001	0.3 (0.2–0.4)	64.9 (48/74)	30.8 (36/117)	<0.001	0.2 (0.1–0.4)
Biochemical pregnancy rate (%)****	13.6 (9/66)	33.1 (88/266)	<0.01	3.1 (1.5–6.6)	5.3 (1/19)	48.0 (24/50)	<0.001	16.6 (2.1–134.2)
Clinical pregnancy rate (%)****	13.6 (9/66)	26.3 (70/266)	<0.05	2.7 (1.1–4.8)	5.3 (1/19)	38.0 (19/50)	<0.01	11.0 (1.4–89.5)
Live birth rate (%)****	6.1 (4/66)	18.4 (49/266)	<0.05	3.5 (1.2–10.0)	0.0 (0/19)	16.0 (8/50)	NS	NA
Miscarriage rate (%)*****	55.6 (5/9)	22.9 (16/70)	NA	NA	100.0 (1/1)	52.6 (10/19)	NA	NA
Preterm birth rate (%)*****	0.0 (0/9)	7.1 (5/70)	NA	NA	0.0 (0/1)	5.3 (1/19)	NA	NA
Congenital malformation rate (%)*****	0.0 (0/9)	0.0 (0/70)	NA	NA	0.0 (0/1)	5.3 (1/19)	NA	NA

Values are presented as mean ± standard deviation or % (n/total), unless otherwise indicated. Statistical significance is set at P<0.05.

FSH, follicle-stimulating hormone; hMG, human menopausal gonadotropin; ET, embryo transfer; NS, not significant; NA, not applicable; CI, confidence interval.

* per number of MII oocytes that were used for ICSI; ** per number of normally fertilized oocytes; *** per number of cycles in which at least two oocytes were obtained; **** per number of ET cycles; ***** per number of clinical pregnancies.

The number of retrieved oocytes per IVF cycle can affect IVF results (21). Accordingly, we compared the developmental outcomes of the three groups (10 or less, 11-15, 16 or more) according to the number of oocytes retrieved per cycle. As a result, we found that AOA increased the fertilization rate and preimplantation embryo developmental rate regardless of the number of retrieved oocytes per IVF cycle (**Supplementary Table 4**).

**Figure 2:**
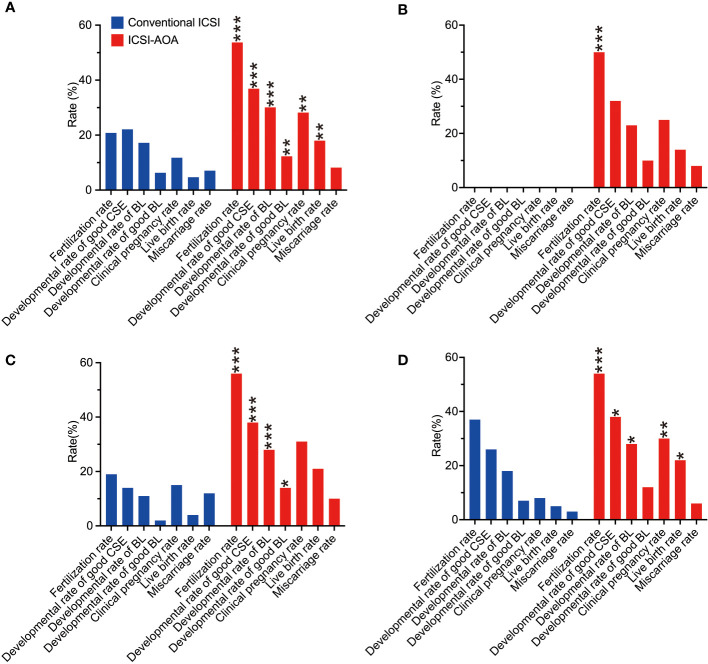
Embryological and clinical outcomes of intracytoplasmic sperm injection (ICSI) and subsequent artificial oocyte activation (AOA) treatment. The patients were distributed into different groups based on their fertilization rates from an initial ICSI cycle. The different categories were: **(A)** all couples (n=198), **(B)** total failed fertilization (0%, n=84), **(C)** low fertilization (>0% to <30%, n=64), and **(D)** moderate fertilization (30% to <50%, n=50). In the total failed fertilization group, p-values could not be calculated for the comparison of developmental outcomes with the ICSI-AOA group because fertilized eggs were not obtained in the 0% fertilization rate group. CSE, cleavage stage embryo; BL, blastocyst stage embryo. *p<0.05, **p<0.01, ***p<0.001.

To clarify the relationship between fertilization rates of conventional ICSI and improvement with AOA, the oocytes were further divided into three groups based on the fertilization rate in previous conventional ICSI cycles (**Figure 2**): 0%, >0%–30%, and >30%–50% groups. ICSI-AOA treatment improved the live birth rate from 0.0% (0/3) to 14.4% (19/132) in the 0% group (NS), 6.5% (2/31) to 21.2% (24/113) in the >0%–30% group (NS), and 4.4% (2/45) to 19.7% (14/71) in the >30%–50% group (OR 5.3, 95% CI 1.1–24.5; *P*<0.05). Because fertilized oocytes were not obtained in the 0% fertilization rate group, p-values could not be calculated for the comparison of developmental outcomes with ICSI-AOA group.

## 4 Discussion

To the best of our knowledge, this multi-center study is the first to use such a large number of oocytes to evaluate the efficacy and safety of ICSI followed by AOA using Ca^2+^ ionophores to increase the low fertilization rates of individual ICSI. Our findings indicate that AOA combined with ICSI improved live birth rate more significantly than ICSI alone. AOA improved all outcomes from fertilization rate to live birth rate, with no known significant adverse perinatal effects. These results are consistent with previous reports (17, 18). Our findings are derived only from couples who experienced fertilization failure, thus excluding male factor infertility that requires surgical treatment. This setting reduced the heterogeneity which was observed in previous studies (22).

Considering that the AOA procedure can be invasive to the oocytes, its indications need to be strictly defined. Factors that affect pregnancy outcome include maternal aging, number of retrieved oocytes, and fertilization rate (21). The search for populations for whom AOA is effective suggested that AOA is particularly likely to be effective in individuals aged ≤ 35 years. In contrast, improvements were reduced in individuals aged ≥ 36 years, suggesting that both sperm and oocyte factors are involved in oocyte activation and that activation factors may decrease during the quality decline that occurs as part of normal oocyte aging (23). In mice in their reproductive age, oocytes required two times more AOA to be activated for parthenogenesis (11); therefore, stronger stimulation with AOA may be effective for older human oocytes.

The efficacy of AOA can be recognized in all groups with fertilization rates <50% after conventional ICSI. In the subgroup analysis divided by fertilization rate of conventional ICSI, there was a trend toward equal embryological and clinical efficacy of ICSI-AOA with fertilization rates ranging from 0-50%.

Previous studies on the efficacy and safety of AOA have focused on male factors (22, 24, 25). In the present study, AOA improved the fertilization and live birth rates, as well as the developmental rate of a good embryo. These results suggest that the mechanism of AOA may be involved in the process of embryonic development and implantation after fertilization (26–28). The signal transduction pathway related to Ca^2+^ stimulation remains largely unexplored (29), requiring further molecular investigation. One possible explanation for these improvements is that AOA may support preimplantation development by induction of zygotic genome activation (ZGA) after fertilization. In fact, somatic cell nuclear transfer studies have shown that inducing ZGA with histone deacetylase inhibitors significantly improves blastocyst development rates, whereas the eight-cell arrest occurs when ZGA does not take place (30, 31). Our results might contribute to the elucidation of the mechanism of the Ca^2+^ signal transduction pathway and of the subsequent embryonic development.

In the current study, regarding the safety of AOA, no congenital abnormalities were observed (61 infants in total), which is consistent with a previous report (32). In contrast, there are concerns that prolonged exposure to A23187 can cause cytoplasmic disruption (33), and that non-physiological increases in Ca^2+^ levels might unexpectedly activate proteins in signal transduction pathways, leading to abnormal gene expressions in long-term prognosis (28, 34). While Capalbo et al. (35) indicated that the use of 10-fold higher concentrations of A23187 does not increase the ratio of chromosome segregation aberrations, the safety of the AOA protocol is still controversial, as lack of proper timing or insufficient duration of AOA may result in premature activation of the oocyte and chromosome segregation aberrations (36). Moreover, it is important to recognize that AOA is an invasive technique involving intervention in fertilized oocytes; as such, it is necessary to establish safe and effective AOA protocols.

Previous reports have made little mention of comparisons between AOA protocols. Our current study showed no significant differences pertaining to pregnancy outcomes among the different AOA protocols. Currently, there is no standard AOA protocol, resulting in a variety of institutions using their own protocols, even in recent studies (17, 37–39). Excessive Ca^2+^ ionophore exposure can be toxic to fertilized oocytes (33); thus, exposure time and Ca^2+^ ionophore concentration should be minimized as much as possible. Further investigation is required to determine appropriate standard AOA protocols.

Some strengths of our present study include the large sample size (the largest at present) and homogeneity in protocols by limiting AOA to the use of Ca^2+^ ionophores. In addition, the oocytes compared in the control and intervention groups were retrieved from the same couples, and a series of treatments from oocyte retrieval to ET was performed at the same institutions, making it possible to compare isogenic populations.

This study has some limitations. First, this was a retrospective cohort study, and a prospective randomized controlled trial is needed to confirm its efficacy. Second, as this study focused on fertilization failure in couples, we did not specify whether the factors of fertilization failure were in the sperm or oocyte. Thus, determining for which factors AOA was most effective in couples experiencing fertilization failure was not possible. A previous study in mice, using the MOAT procedure, reported that ICSI-AOA treatment improved the live birth rate for fertilization failure caused by sperm-related factors greater than that for failure caused by oocyte-related factors (8). Technology that can detect the cause of fertilization failure may allow for more personalized treatment (40). Meanwhile, it has been suggested that AOA may be effective for both sperm and oocyte factor fertilization failure (8). It is necessary to explore a wider range of couples who may benefit from AOA, not limited to sperm-related factors. Thirdly, some of the outcomes in the subgroup analysis were limited by the small sample size (ex. miscarriage rate, preterm birth rate or congenital malformation rate in the conventional ICSI group). When divided based on the fertilization rate in previous conventional ICSI cycles, p-values could not be calculated for the comparison of developmental outcomes with ICSI-AOA group because fertilized eggs were not obtained in the 0% fertilization rate group. Even with the selection of an appropriate statistical analysis, a relatively small sample size may result in a lack of statistical power. Finally, as the information was obtained from multiple institutions, there were variations in ovarian stimulation, AOA protocols, and the stage and grade of transferred embryos among the institutions. Although a previous study reported that live birth rate is not affected by gonadotropin dose or duration of ovarian stimulation, and that there is no significant difference in clinical pregnancy rate or live birth rate between the different AOA protocols (41), the possibility of some bias cannot be ruled out.

In clinical practice, AOA may be the final option for couples experiencing fertilization failure. Despite the results of many outstanding studies, the efficacy and safety of AOA have not yet been established. We showed that ICSI-AOA using Ca^2+^ ionophores significantly improved the live birth rate of couples whose previous fertilization rate in conventional ICSI was ≤ 50%. Women who are younger and have no history of oocyte retrieval were especially more likely to benefit from AOA. Those who receive AOA treatment might expect similar improvements regardless of the fertilization rate with former conventional ICSI. This study may be an important decision-making tool when considering the implications of AOA. The results of our study could lead to increasing clinical applications of AOA using Ca^2+^ ionophores following ICSI, with a subsequent increase in live births. The efficacy and safety of AOA should be evaluated using a unified AOA protocol, and the population for whom AOA is most effective requires further investigation.

## Data availability statement

The original contributions presented in the study are included in the article/**Supplementary Material**. Further inquiries can be directed to the corresponding author.

## Ethics statement

The studies involving human participants were reviewed and approved by the institutional research ethics board of Keio University School of Medicine (approval number: 20211097). Written informed consent for participation was not required for this study in accordance with the national legislation and the institutional requirements.

## Author contributions

KA and MY designed the study and performed data analysis and interpretation with HU, SK, and NK. KA and MY wrote the manuscript with input from all authors. KA and SJ performed the statistical analysis. All authors contributed to the article and approved the submitted version.
