# Supplementary material for: Artificial oocyte activation using Ca2+ ionophores following intracytoplasmic sperm injection for low fertilization rate
Source: Front Endocrinol (Lausanne). 2023 Mar 9;14:1131808. doi: 10.3389/fendo.2023.1131808 (PMC10034378; doi:10.3389/fendo.2023.1131808)
Supplement: Supplementary file 1 [file Table_1.docx]

Supplementary Material

Artificial oocyte activation using Ca^2+^ ionophores following intracytoplasmic sperm injection for low fertilization rate

**Kazuhiro Akashi,** **Mitsutoshi Yamada*, Seung Chik Jwa, Hiroki Utsuno, Shintaro Kamijo, Yasushi Hirota, Mamoru Tanaka, Yutaka Osuga, Naoaki Kuji**

*** Correspondence:**

Mitsutoshi Yamada

[mitsutoshi.yamada@gmail.com](mailto:mitsutoshi.yamada@gmail.com)

# Supplementary Figures and Tables

**Supplementary Table 1. Baseline characteristics of the included females**

| **Characteristics** | |  |
| --- | --- | --- |
| Patients (n) | | 198 |
| Female age at the initial visit (years) | | 35.3 ± 3.8 |
| Period of infertility (months) | | 38.3 ± 38.2 |
| BMI (kg/m^2^) | | 21.5 ± 3.1 |
| AMH (ng/mL) | | 3.2 ± 2.8 |
| Antral follicle count | | 8.9 ± 6.3 |
| Basal FSH (IU/L) | | 7.4 ± 2.9 |
| History of miscarriage (n) | 0 | 152 |
|  | 1 | 32 |
|  | <2 | 14 |
| History of live births (n) | 0 | 176 |
|  | 1 | 19 |
|  | <2 | 3 |
| History of smoking (n) | Yes | 22 |
|  | No | 156 |
|  | Unknown | 20 |
| Indication for ART (n) | Tubal infertility | 62 |
|  | Male infertility | 105 |
|  | Endometriosis | 16 |
|  | Unexplained | 56 |
| Number of in vitro fertilization treatment cycles before the initial visit | 0 | 155 |
|  | <1 | 43 |

Values are presented as mean ± standard deviation, unless otherwise indicated.

BMI, body mass index; AMH, anti-Mullerian hormone; FSH, follicle stimulating hormone; ART, assisted reproductive technology

**Supplementary Table 2. Comparison of ovarian stimulation protocols**

|  | **Natural cycle (%)** | **hMG/FSH + CC (%)** | **GnRH agonist, hMG/FSH (%)** | **GnRH antagonist, hMG/FSH (%)** | **PPOS (%)** | **Others (%)** |
| --- | --- | --- | --- | --- | --- | --- |
| **Clinical pregnancy rate** | 30.0 (6/20) | 29.7 (11/37) | 23.6 (29/123) | 26.4 (42/159) | 100.0 (1/1) | 16.4 (10/61) |
| **Live birth rate** | 25.0 (5/20) | 13.5 (5/37) | 15.4 (19/123) | 15.7 (25/159) | 100.0 (1/1) | 9.8 (6/61) |

The data represent the clinical pregnancy and live birth rates per embryo transfer. They were not significantly different among all protocols.

CC, clomiphene citrate; hMG, human menopausal gonadotropin; FSH, follicle-stimulating hormone; GnRH, gonadotropin-releasing hormone; PPOS, progestin-primed ovarian stimulation

**Supplementary Table 3. Comparison of embryo transfer outcomes between the two Ca^2+^ ionophores**

| **AOA protocol** | **A23187** | **Ionomycin** | ***P*-value** |
| --- | --- | --- | --- |
| **Clinical pregnancy rate** | 28.1% (81/288) | 28.6% (8/28) | NS |
| **Live birth rate** | 18.1% (52/288) | 17.9% (5/28) | NS |

Values are presented as % (n/total). There were no significant differences in the clinical pregnancy and live birth rates between both methods.

AOA, artificial oocyte activation

**Supplementary Table 4. Embryological outcomes after intracytoplasmic sperm injection (ICSI) and subsequent artificial oocyte activation (AOA) treatment distributed based on the number of oocytes per retrieval cycle**

|  | **10 or less retrieved oocytes per cycle** | | | **11-15 retrieved oocytes per cycle** | | | **16 or more retrieved oocytes per cycle** | | |
| --- | --- | --- | --- | --- | --- | --- | --- | --- | --- |
|  | **Conventional ICSI** | **ICSI-AOA** | **p** | **Conventional ICSI** | **ICSI-AOA** | **p** | **Conventional ICSI** | **ICSI-AOA** | **p** |
| **Fertilization rate*** | 17.70% | 56.00% | <0.001 | 26.00% | 55.00% | <0.001 | 22.80% | 47.40% | <0.001 |
|  | (128/725) | (561/1002) |  | (78/300) | (236/429) |  | (79/347) | (219/462) |  |
| **Developmental rate of good cleavage stage embryo**** | 21.90% | 37.40% | <0.001 | 25.60% | 35.60% | NS | 19.00% | 37.00% | <0.01 |
|  | (28/128) | (210/561) |  | (20/78) | (84/236) |  | (15/79) | (81/219) |  |
| **Developmental rate of blastocyst embryo**** | 14.80% | 28.00% | <0.05 | 21.80% | 30.90% | NS | 16.50% | 34.70% | <0.01 |
|  | (19/128) | (157/561) |  | (17/78) | (73/236) |  | (13/79) | (81/219) |  |
| **Developmental rate of good blastocyst embryo**** | 5.50% | 11.10% | NS | 11.5% (9/78) | 15.30% | NS | 2.50% | 12.30% | <0.05 |
|  | (7/128) | (62/561) |  |  | (36/236) |  | (2/79) | (27/219) |  |

Values are presented as mean ± standard deviation or % (n/total), unless otherwise indicated. Statistical significance is set at *P<*0.05.

ET, embryo transfer; NS, not significant; NA, not applicable; CI, confidence interval

* per number of MII oocytes that were used for ICSI; ** per number of normally fertilized oocytes

**
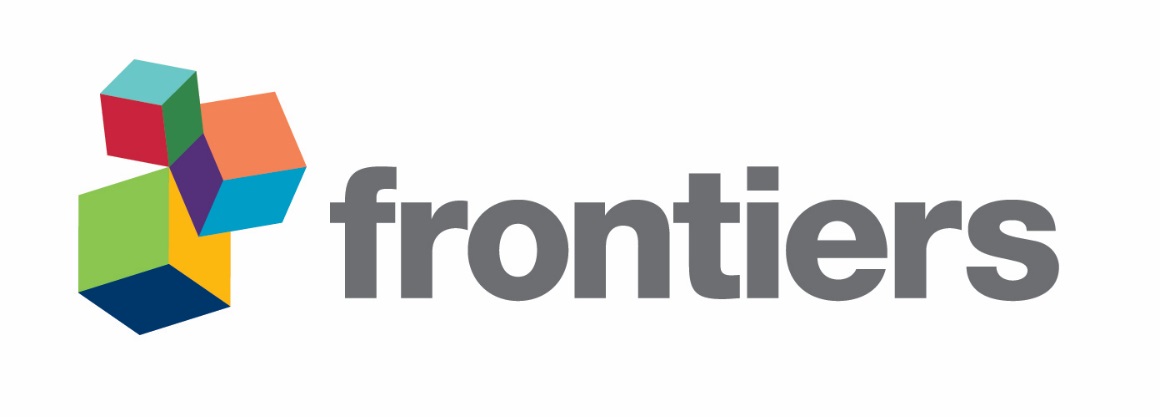
**
